# The Role of Oxidative Stress in the Development of Diabetes Mellitus and Its Complications

**DOI:** 10.1155/2019/4189813

**Published:** 2019-05-05

**Authors:** Julia M. Dos Santos, Shikha Tewari, Roberta H. Mendes

**Affiliations:** ^1^School of Education, Health and Human Performance, Fairmont State University, West Virginia, USA; ^2^Henry Ford College, Dearborn, Michigan, USA; ^3^Department of Pathology, King George Medical University, Lucknow, India; ^4^UCD School of Agriculture and Food Science Institute of Food and Health, University College Dublin, Belfield, Dublin 4, Ireland

The thematic issue addresses the role of oxidative stress in the development of diabetes and its related complications. Diabetes is a growing pandemic of the 21st century that has reached 1 in every 11 people worldwide [1]. Changes in lifestyle such as unhealthy diet and physical inactivity are strongly associated with the growing prevalence of this disease [2, 3]. Chronic hyperglycemia, the common outcome of all diabetes types, may negatively influence the structure and function of many organ systems, particularly the cardiovascular, nervous, and renal systems [4, 5]. These diabetic complications are associated with increased rate of morbidity and mortality [1, 5]. The role of oxidative stress has been an important piece of the puzzle for the understanding of the complex mechanism by which diabetes and its complications are developed. In this context, numerous research groups have focused on the characterization of the reactive oxygen species (ROS) source, its triggered pathway, scavenging and antioxidant substances in diabetes [6–9]. The multifaceted effects of oxidative damage in diabetes have been well addressed in the current issue and it provides a bigger picture of complication in a single platform. Therefore, this special issue includes 8 research articles focusing on the role of oxidative stress and antioxidant defense on the development of diabetes and its associated diseases. The guest editors are pleased to present a compendium of these cutting-edge original research and review articles as follows.

In the research article “Astragalus membranaceus and Panax notoginseng, the Novel Renoprotective Compound, Synergistically Protect against Podocyte Injury in Streptozotocin-Induced Diabetic Rats,” ever since the diabetic complications have been understood and oxidative injury has been established as a cause, many compounds/plant extracts that attenuate oxidative damage and cell death have been researched upon. Adding to this field, one article in this issue showed the renoprotective role of treatment with Astragalus membranaceus (AG) and Panax notoginseng (NG). Treatment significantly reduced albuminuria and improved renal histopathology and podocyte foot process effacement in STZ-induced diabetic rats. AG and NG synergistically attenuated the structural and functional abnormalities in the kidney, and in the future, it may provide treatment combination for diabetic nephropathy and other kidney diseases.

The research article “Myocardial Ischemia and Diabetes Mellitus: Role of Oxidative Stress in the Connection between Cardiac Metabolism and Coronary Blood Flow” discusses the physiological and pathophysiological role of oxidative stress in myocardial metabolism and coronary blood flow (CBF), with particular attention to patients with diabetes mellitus. Ischemic heart disease (IHD) being one major current health risk to diabetic patients, the review summarizes the role of oxidative stress in coronary microvascular dysfunction and development of IHD. Oxidative stress further generates ROS, AGEs, and oxidized LDL causing coronary microvascular dysfunction. Therein inflammatory pathway is triggered and ion channel function is altered. The review suggests that balancing the oxidative stress and restoring the ion channel function may be exploited as therapeutic target in the treatment of IHD.

In the research article “Effect of Baoshenfang Formula on Podocyte Injury via Inhibiting the NOX-4/ROS/p38 Pathway in Diabetic Nephropathy,” it is stated that more and more studies demonstrated that diabetic nephropathy is the leading cause of end-stage of renal diseases [10]. Previous study has demonstrated that increased ROS plays a key role in podocyte injury in diabetic nephropathy (DN) [11, 12]. NOX-4, as an important number of nicotinamide adenine dinucleotide phosphate (NADPH) oxidase, is the major source of ROS production in podocyte. This study evaluated the effects of Baoshenfang (BSF: Chinese herbal formula) on podocyte injury *in vivo* and *in vitro* and explored the possible involvement of the nicotinamide adenine dinucleotide phosphate-oxidase-4/reactive oxygen species- (NOX-4/ROS-) activated p38 pathway. The BSF shows therapeutic potential in attenuating oxidative stress and apoptosis in podocytes in DN.

In the research article “Evidence in Practice of Tissue Healing with Latex Biomembrane: Integrative Review,” wound healing is a perfectly coordinated cascade of cellular, molecular, and biochemical events which interact in tissue reconstitution. Chronic diseases such as pressure ulcers (PU) and diabetes mellitus (DM) are considered risk factors for wound healing. Latex biomembrane (LBM), a biocompatible material, derived from latex of rubber tree (*Hevea brasiliensis*) appears to create tendencies as an angiogenic inducing tissue healing agent and as biomaterial, resulting from its structural qualities and its low cost when compared to conventional treatments. Therefore, the authors aim to summarize the results, methods, and scientific findings that certify or recommend the use of LBM as a new technique to be applied effectively in the treatment of wounds.

In the research article “Glucagon-Like Peptide-1 Receptor Agonist Protects Dorsal Root Ganglion Neurons against Oxidative Insult,” diabetic polyneuropathy (PDN) is one of the most prevalent complications in diabetes. In the article from this issue, exendin-4, a glucagon-like peptide 1 receptor agonist (GLP-1RA), was shown beneficial against oxidative stress insults by activating superoxide dismutase in dorsal root ganglion neuron cell. Therefore, the authors suggest that GLP-1RA attenuates PDN by protecting insults on dorsal root ganglion neuron cell.

In the research article “Effects of High-Fat Diet on eHSP72 and Extra-to-Intracellular HSP70 Levels in Mice Submitted to Exercise under Exposure to Fine Particulate Matter,” tha authors suggest that in type 2 diabetes, eHSP70 has been negatively correlated with iHSP70 in skeletal muscles and that leds to impaired glucose uptake. The elevated levels of eHSP70 are associated with insulin resistance in elderly population [13]. In this issue, we discuss an interesting finding correlating two major global health issues of obesity and air pollution (fine particulate matter PM2.5), with reference to diabetes, demonstrating that HFD impaired exercise performance and weakened the standard heat shock response to exercise. This is a concerning fact that regular exercise abrogates oxidative stress and modulates many pathways improving insulin resistance and glucose uptake in skeletal muscle [3], but the growing worldwide pollution may weaken the response.

In the research article “A New Way for Beta Cell Neogenesis: Transdifferentiation from Alpha Cells Induced by Glucagon-Like Peptide 1,” it is stated that current treatment modalities for diabetes include drug therapy or pancreatic islet transplantation which has its own limitations [14]. One study in the current issue highlights the use of glucagon-like peptide 1 (GLP1), a gut-derived hormone secreted by the intestine as a prospective target for type 2 diabetes therapy. It is interesting to discuss how it ameliorates hyperglycemia in diabetic models, inducing regeneration of beta cells by the endogenous neogenesis in a rat model. This study elaborates cellular and molecular mechanisms that regulate adult pancreatic differentiation in the management of diabetes.

In the research article “Advanced Glycation End Products Increase MDM2 Expression via Transcription Factor KLF5,” the authors state that increase in the level of AGEs has been associated with diabetic complications for a long time; however, their role in the overexpression of MDM2 and colon cancer development is an exciting finding by one of the papers in the current issue. The research highlights the activation of oncogenic pathway through activation of MDM causing Rb and p53 degradation, which may stimulate cell proliferation and metastasis. The concept of initiation of tumorigenesis or epithelial mesenchymal transition, in diabetics, via P53, AKT/mTOR, RAS signaling or Wnt signaling is a recent an open platform to explore.

The editors anticipate this special issue to be of tremendous interest to the medical and scientific community. We hope researchers benefit in this issue to continuously make further progress in the understanding of the role of oxidative stress in the development of diabetes and its complications.
